# Hyperspectral Imaging-Based Rapid Assessment of Chinese Yam Quality for Dried Slice Processing

**DOI:** 10.3390/foods15173133

**Published:** 2026-09-03

**Authors:** Tingting Shen, Yang Yang, Dou Yang, Chaoyi Chen, Xiaodong Zhai, Zhihua Li, Junjun Zhang, Roujia Zhang, Tianxing Wang, Xiaowei Huang, Jiyong Shi

**Affiliations:** School of Food Science and Engineering, Jiangsu University, Zhenjiang 212013, China; shentingtingstt@ujs.edu.cn (T.S.); 2212418044@stmail.ujs.edu.cn (Y.Y.); yangdou0121@163.com (D.Y.); 18985206970@163.com (C.C.); zhai_xiaodong@ujs.edu.cn (X.Z.); lizh@ujs.edu.cn (Z.L.); junjun_5457@ujs.edu.cn (J.Z.); 1000005191@ujs.edu.cn (R.Z.); ujs-tx.wang@ujs.edu.cn (T.W.); huangxiaowei@ujs.edu.cn (X.H.)

**Keywords:** Chinese yam, dried yam slices, hyperspectral imaging, chemometrics, quality assessment, texture

## Abstract

Chinese yam (*Dioscorea* spp.) is widely used as food and traditional medicine, but varietal differences may affect dried yam slice quality. This study compared ten Chinese yam varieties, examined relationships between raw-material quality indicators and dried yam slice quality, and evaluated hyperspectral imaging for rapid assessment before processing. Significant varietal differences were observed in reducing sugar content, total phenolic content, and texture properties (*p* < 0.05), and trait–quality relationships were variety-dependent. Spectral preprocessing, variable selection, and regression modelling were used to predict the reference values of reducing sugar content and total phenolic content obtained using the specified analytical procedures, together with fresh-slice hardness. The best models combined principal component analysis with decision tree regression (PCA-DTR), competitive adaptive reweighted sampling with partial least squares regression (CARS-PLSR), and CARS with random forest regression (CARS-RFR), respectively. Prediction-set coefficients of determination ($R_{P}^{2}$) were 0.9891, 0.9335, and 0.9314, with root mean square errors of prediction (RMSEP) of 0.0981%, 0.0905 mg gallic acid equivalents/100 g dry weight, and 130.3973 gf, and residual predictive deviation (RPD) values of 9.7535, 3.9438, and 3.8820, respectively. These results support hyperspectral imaging with chemometrics for rapid assessment and selection of yam raw materials for dried yam slice processing.

## 1. Introduction

Yam tubers (*Dioscorea* spp.) are widely used as both food and traditional medicine because of their nutritional value and bioactive components [1]. More than 600 *Dioscorea* species have been reported worldwide, with numerous species and local varieties occurring in China [2]. Commonly consumed yam types, including Huai yam, purple yam, and iron-stick yam, differ in their physicochemical characteristics and processing properties [3,4]. However, fresh yam has a high moisture content and is susceptible to quality deterioration during storage and transportation. It is therefore commonly processed into products such as dried yam slices and yam powder to extend shelf life and improve its utilization [5,6]. The physicochemical and textural characteristics of fresh yam tubers may affect their behaviour during processing and the quality of the resulting dried products. Differences among yam varieties in chemical composition and tissue characteristics may also lead to different responses during drying and variations in dried slice quality [7]. Previous studies on dried yam slices have mainly examined the effects of drying methods, temperatures, and pretreatments on drying characteristics, colour, texture, rehydration properties, microstructure, and nutrient retention [8,9,10,11]. However, the relationships between the raw material quality traits of different fresh yam varieties and the quality of the corresponding dried slices remain insufficiently understood, limiting the scientific selection of raw materials for dried slice processing.

Reducing sugar content, total phenolic content, and textural properties are important quality indicators for evaluating the suitability of yam raw materials for dried yam slice processing. Reducing sugars can participate in non-enzymatic browning reactions during drying and may therefore contribute to colour development and quality changes in dried products [12]. Total phenolic content is an important indicator of the phenolic composition and antioxidant potential of yam tubers, and changes in phenolic compounds during processing may influence the nutritional characteristics of dried products [13]. In addition, textural properties, including hardness, cohesiveness, and adhesiveness, reflect the mechanical and structural characteristics of fresh yam tissues and may be associated with structural changes during drying and the final texture of dried slices [14,15]. Previous studies have reported variations in physicochemical characteristics and quality attributes among different yam varieties and yam-based products. However, systematic investigations linking the physicochemical and textural quality indicators of yam raw materials from multiple varieties with the quality of their corresponding dried slices remain limited. Clarifying these relationships could provide a scientific basis for raw material selection and quality evaluation for dried yam slice processing.

Conventional chemical methods for determining reducing sugar and total phenolic contents are generally reliable and provide reference values for model development; however, they usually require sample preparation, chemical reagents, and multiple analytical steps [16,17]. Instrumental texture measurements commonly involve direct mechanical loading, such as compression or penetration, which may deform or damage the sample during testing [18]. Although these methods provide useful reference measurements, they are time-consuming and may consume samples or require direct mechanical testing, making them difficult to apply to the rapid evaluation of large batches of yam raw materials. In practical processing, particularly when different yam varieties or production batches need to be screened before drying, a rapid method for assessing raw material quality is therefore needed.

Hyperspectral imaging (HSI) is a rapid, non-contact analytical technique that integrates spectral and spatial information. Because it can capture spectral responses associated with chemical composition and tissue structure, HSI has been widely applied to quality evaluation, geographical origin identification, and component prediction in food and agricultural products [19]. However, applications of HSI to yam quality assessment remain relatively limited. Zhang et al. [20] combined HSI with chemometric methods for the rapid identification of yam powder samples and the estimation of the contents of index components, whereas Adesokan et al. [21] applied HSI and machine learning models to estimate the dry matter content of whole fresh yam tubers. HSI has also been used to predict chemical composition and texture-related quality attributes in other agricultural products. For instance, Wang et al. [22] used HSI to predict acidity, sugar content, and hardness in navel oranges, while Ahmed et al. [23] combined HSI with explainable deep learning models to predict sweet potato firmness. Collectively, these studies indicate the potential of HSI for rapid quality assessment of agricultural products and raw materials. Nevertheless, although hyperspectral imaging has been increasingly investigated for nondestructive quality evaluation of agricultural and food products [24], HSI-based studies focusing on raw-material quality indicators relevant to dried yam slice processing, particularly reducing sugar content, total phenolic content, and hardness, remain scarce.

The prediction performance of HSI models depends not only on the acquired spectral information but also on spectral preprocessing, dimensionality reduction, characteristic wavelength selection, and regression algorithms. Spectral preprocessing is commonly used to reduce noise, baseline drift, and scattering effects, whereas dimensionality reduction and wavelength selection can reduce redundant spectral variables and improve modelling efficiency [25]. Principal component analysis (PCA) is commonly used for spectral dimensionality reduction, whereas competitive adaptive reweighted sampling (CARS) and the successive projections algorithm (SPA) are frequently used to select informative wavelengths. Gao et al. [26] combined HSI with CARS, SPA, PCA, and partial least squares regression to predict the soluble solids content of Red Globe grapes, demonstrating the importance of spectral variable processing in hyperspectral quality prediction. In addition, regression models such as partial least squares regression (PLSR), support vector regression (SVR), random forest regression (RFR), and decision tree regression (DTR) can model different linear and nonlinear relationships between spectral variables and reference measurements [27]. Therefore, comparing different combinations of spectral preprocessing, variable selection, and regression models is useful for identifying suitable models for the rapid assessment of yam quality attributes.

Accordingly, this study aimed to: (1) characterize and compare selected compositional and texture-related quality indicators among ten Chinese yam varieties; (2) determine the relationships between these raw-material quality indicators and the quality of the corresponding dried yam slices; and (3) develop and compare HSI-based prediction models using spectra acquired from fresh yam slices for the reference values of reducing sugar content and total phenolic content obtained using specified analytical procedures, together with fresh-slice hardness. The findings may provide a basis for the rapid assessment and selection of yam raw materials before dried yam slice processing.

## 2. Materials and Methods

### 2.1. Sample Collection and Preparation

Ten fresh yam varieties from different production areas in China were used in this study: Huai yam (Jiaozuo, Henan, China), Yulong Snow Mountain glutinous rice yam (Lijiang, Yunnan, China), Yunnan glutinous rice yam (Yuxi, Yunnan, China), Xishi seed yam (Heze, Shandong, China), Xiaobaizui white yam (Baoding, Hebei, China), purple yam (Ganzhou, Jiangxi, China), Buddha’s hand yam (Huanggang, Hubei, China), Wencheng glutinous rice yam (Wenzhou, Zhejiang, China), iron stick yam (Jiaozuo, Henan, China), and vegetable yam (Zhenjiang, Jiangsu, China). The varieties were numbered from 1 to 10 in the order listed above. Fresh yam tubers with similar size and appearance and without visible mechanical damage, mold, decay, pests, or disease symptoms were selected for the experiments.

The selected tubers were stored in a refrigerator at 4 °C until sample preparation. Before preparation, the tubers were removed from the refrigerator and allowed to equilibrate to room temperature to reduce temperature-related spectral variation and surface condensation during subsequent hyperspectral imaging and texture measurements. The tubers were then washed, peeled, and cut into 4-mm-thick slices using a stainless-steel knife. The knife was washed with water and wiped dry before processing each variety to minimize cross-contamination. For each variety, 12 individual yam tubers were used. One 4-mm-thick slice from each tuber was used for hyperspectral imaging, resulting in 12 hyperspectral samples per variety and 120 samples in total. Adjacent slices from the same tuber were used for physicochemical analyses and texture measurements so that the spectral data and reference measurements corresponded to the same individual tuber. Accordingly, 12 spectral observations were obtained for each variety, giving a total of 120 observations for subsequent model development and statistical analysis.

### 2.2. Determination of Reducing Sugar Content

The reducing sugar content used as the chemical reference value for subsequent HSI modelling was determined using the 3,5-dinitrosalicylic acid (DNS, Sinopharm Chemical Reagent Co., Ltd., Shanghai, China) colorimetric method with slight modifications [17]. Corresponding yam slices from the same tubers were dried at 60 °C for 8 h, ground into powder, sieved, and stored in a desiccator as part of the standardized analytical sample-preparation procedure. Yam powder (0.5 g) was extracted with distilled water in a water bath at 50 °C for 30 min. The extract was centrifuged at 4000 rpm for 5 min, and the supernatant was collected. The residue was washed once with distilled water and centrifuged again under the same conditions. The combined supernatants were diluted to 100 mL with distilled water.

For color development, 1 mL of the extract was mixed with 2 mL of DNS reagent and heated in a boiling water bath for 5 min. After cooling to room temperature, 4 mL of distilled water was added, and the absorbance was measured at 540 nm using a UV–visible spectrophotometer (Sinopharm Chemical Reagent Co., Ltd.). Glucose (Sinopharm Chemical Reagent Co., Ltd., Shanghai, China) was used as the standard, and the reducing sugar content was calculated from the calibration curve and expressed as a percentage (%).

### 2.3. Determination of Total Phenolic Content

The total phenolic content (TPC) used as the chemical reference value for subsequent HSI modelling was determined using the Folin–Ciocalteu colorimetric method with modifications based on Guo et al. [16]. The yam powder used for TPC determination was prepared from the corresponding samples using the same drying and grinding procedure described in Section 2.2. Gallic acid (Sinopharm Chemical Reagent Co., Ltd., Shanghai, China) was used as the standard, and absorbance was measured at 765 nm.

Yam powder (2.0 g) was extracted with 70% ethanol (Sinopharm Chemical Reagent Co., Ltd., Shanghai, China) at a solid-to-liquid ratio of 1:4 (g/mL) under ultrasonic treatment at 30 °C for 30 min. The extract was centrifuged at 8000 rpm for 12 min, and the supernatant was collected for analysis.

For color development, 1 mL of the extract was mixed with 2 mL of distilled water and 1.5 mL of Folin–Ciocalteu reagent (Shanghai yuanye Bio-Technology Co., Ltd., Shanghai, China), followed by the addition of 1.5 mL of 10% Na_2_CO_3_ solution (Sinopharm Chemical Reagent Co., Ltd., Shanghai, China). The mixture was kept at room temperature in the dark for 2 h, and the absorbance was measured at 765 nm using a UV–visible spectrophotometer. A reagent blank was used as the control, and all measurements were performed in triplicate. The TPC was calculated as follows:

(1) $$TPC=\frac{C\times V\times N}{10\times m}$$

where TPC is expressed as mg gallic acid equivalent per 100 g dry weight sample (mg GAE/100 g DW); *C* is the gallic acid equivalent concentration of the sample extract calculated from the calibration curve (μg/mL); *V* is the volume of the extract (mL); *N* is the dilution factor; and *m* is the sample mass (g).

### 2.4. Texture Measurement of Fresh Yam Slices

The texture properties of fresh yam slices were measured using a TA.XT Plus texture analyzer (Stable Micro Systems, Surrey, UK) based on the method of Li et al. [28], with modifications. Texture profile analysis (TPA) was performed using a 6 mm cylindrical probe (P/6). The pre-test, test, and post-test speeds were all set at 1.0 mm/s, and the trigger force was 3 g. Each sample was subjected to two consecutive compression cycles. For each yam variety, 12 slices were measured independently. Hardness, cohesiveness, and adhesiveness were calculated from the resulting force–time curves. Cohesiveness was calculated as a dimensionless ratio. Adhesiveness was expressed as the negative area of the force–time curve, with more negative values indicating stronger adhesion.

### 2.5. Texture Measurement of Dried Yam Slices

Fresh yam slices prepared as described in Section 2.1 were dried at 60 °C for 8 h and then cooled to room temperature in a desiccator. The dried slices were tested using the same texture analyzer, probe, test speeds, and trigger force described in Section 2.4. For each yam variety, 12 dried slices were measured independently. The maximum force recorded during the first compression cycle was used to characterize the hardness of the dried slices. Cohesiveness and adhesiveness were also obtained from the force–time curves.

### 2.6. Hyperspectral Image Acquisition and Spectral Extraction

**Figure 1:**
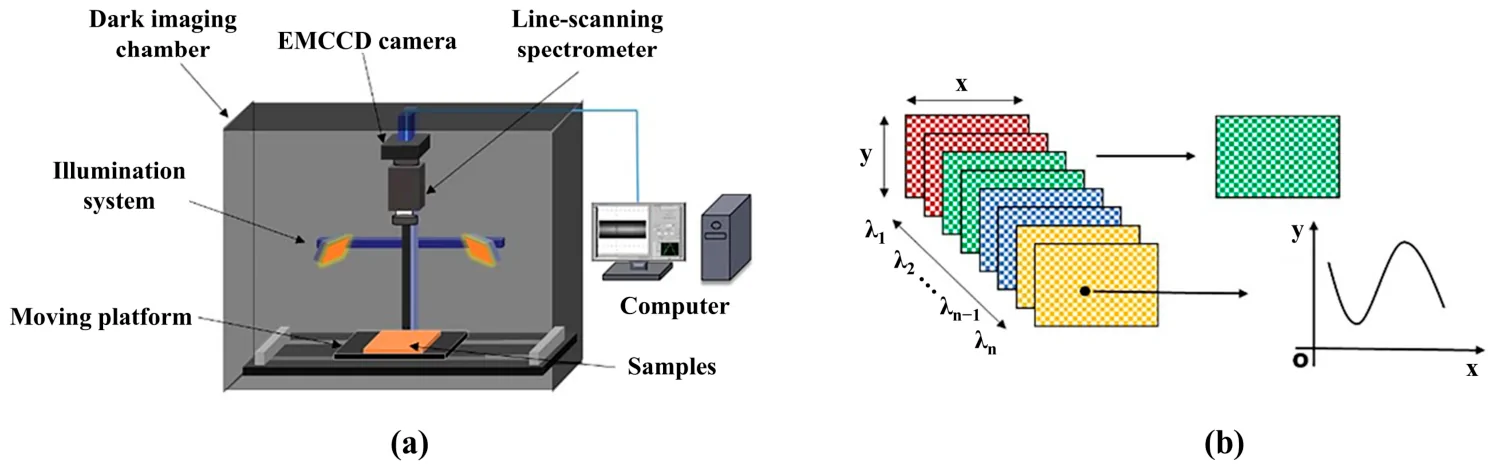
Schematic diagram of hyperspectral image acquisition for fresh yam slices: (**a**) line-scan hyperspectral imaging system; (**b**) hyperspectral data cube.

The hyperspectral images of fresh yam slices were acquired using a line-scan hyperspectral imaging system in reflectance mode, as shown in Figure 1a. The system consisted of a dark imaging chamber, an EMCCD camera (Raptor EM285CL, Raptor Photonics Ltd., Larne, UK), an imaging spectrograph (ImSpector V10E, Spectral Imaging Ltd., Oulu, Finland), a 150 W illumination system (Fiber-Lite PL900, Dolan-Jenner Industries Inc., Boxborough, MA, USA), an electronically controlled moving platform (Zolix TS200AB, Zolix Corp., Beijing, China), a computer (HPdx2390MT, Hewlett-Packard, Beijing, China), and image acquisition and analysis software (Spectral Image, Isuzu-Optics Co., Hsinchu, Taiwan).

Each hyperspectral image was saved as a three-dimensional hypercube containing two spatial dimensions and one spectral dimension, as illustrated in Figure 1b. The spectra used for subsequent analysis covered approximately 371–1028 nm and contained 616 spectral bands.

Before image acquisition, the illumination system was turned on and allowed to stabilize for 30 min. The yam slices were placed on a black background plate on the moving platform. The focus and platform speed were adjusted to obtain clear hyperspectral images. White and dark reference images were collected before sample scanning to correct for uneven illumination and dark current. The corrected reflectance image was calculated as follows:

(2) $$R_{\lambda }=\frac{I_{\lambda }-B_{\lambda }}{W_{\lambda }-B_{\lambda }}$$

where $R_{\lambda }$ is the corrected reflectance at wavelength λ; $I_{\lambda }$ is the original intensity of the sample image; $B_{\lambda }$ is the dark reference intensity; and $W_{\lambda }$ is the white reference intensity.

The corrected hyperspectral images were imported into ENVI 5.6 software for spectral extraction. For each yam slice, the sample region was selected as the region of interest (ROI), avoiding the background and obvious edge interference. The spectra of all pixels within the ROI were averaged to obtain one mean spectrum for each slice. A total of 120 mean spectra were obtained for subsequent spectral preprocessing and model development.

### 2.7. Spectral Preprocessing and Sample Partitioning

The mean spectra extracted from the regions of interest were used for model development. To reduce spectral noise, baseline variation, and scattering-related effects, several preprocessing methods were compared, including multiplicative scatter correction (MSC), standard normal variate (SNV) transformation, Savitzky–Golay (SG) smoothing, SG combined with SNV, SG combined with MSC, moving average smoothing, first derivative, second derivative, wavelet transform, mean centralization, standardization, and min–max normalization [29]. SG smoothing was performed using an 11-point window and a second-order polynomial. For SG+SNV preprocessing, SG smoothing was followed by sample-wise SNV transformation. The preprocessing performance was evaluated through preliminary PLSR modelling, and SG+SNV was selected for subsequent characteristic wavelength selection and model development.

For each target variable, the 120 samples, each representing a different individual tuber, were separately divided into a calibration set and a prediction set using the sample set partitioning based on joint X–Y distances (SPXY) algorithm [30]. A calibration-to-prediction ratio of 3:1 was adopted, resulting in 90 samples in the calibration set and 30 samples in the prediction set for each target variable. SPXY considers sample differences in both the spectral (X) space and the corresponding reference-value (Y) space during sample partitioning. Because reducing sugar content, total phenolic content, and hardness represented different response variables, SPXY partitioning was performed separately for each target variable. Because each tuber contributed only one hyperspectral observation, no individual tuber was represented in both the calibration and prediction sets within a given target-specific partition. The calibration set was used for model fitting and parameter setting, whereas the prediction set was reserved exclusively for final model evaluation and was not involved in model training or parameter optimization.

### 2.8. Spectral Feature Selection and Dimensionality Reduction Procedures

To reduce redundant spectral variables and improve modelling efficiency, dimensionality reduction and feature selection were performed before regression modelling. Three methods were compared, including PCA, CARS, and SPA. PCA was used to extract representative spectral components from the preprocessed full spectra, whereas CARS and SPA were used to select informative wavelengths from the preprocessed spectral variables [31,32].

The selected components or wavelengths were then used as input variables for subsequent regression models.

### 2.9. Model Development and Evaluation

Quantitative prediction models were developed using the retained principal components or selected characteristic wavelengths as input variables. Four regression models were compared, including partial least squares regression (PLSR), support vector regression (SVR), random forest regression (RFR), and decision tree regression (DTR). SVR was implemented using a radial basis function kernel with C = 10, ε = 0.01, and γ set to “scale”. RFR consisted of 100 decision trees, with the number of features considered at each split set to the square root of the total number of input features. For DTR, the maximum tree depth was set to 10 and the minimum number of samples per leaf was set to 2. For PLSR, the numbers of latent variables used for PCA-, CARS-, and SPA-based inputs were 3, 13, and 8 for reducing sugar content; 3, 10, and 9 for total phenolic content; and 6, 9, and 8 for hardness, respectively. When required by the corresponding algorithm, the input variables were standardized before model training.

For reducing sugar and TPC modelling, the response variables were the chemical reference values obtained from the corresponding samples using the sample-preparation and analytical procedures described in Section 2.2 and Section 2.3. Hardness, in contrast, was measured directly on fresh yam slices. The hyperspectral data used for all three prediction targets were acquired from fresh slices before drying. For each quality indicator, models were established using the corresponding calibration set and evaluated using the corresponding prediction set. Within each target variable, the same calibration and prediction subsets were used for all dimensionality-reduction/feature-selection methods and regression models to ensure consistent model comparison. The prediction set was not used for model fitting or parameter optimization. Because reducing sugar content, total phenolic content, and hardness may be associated with different spectral variables and modelling characteristics, the best-performing model was selected separately for each quality indicator.

The performance of the models was evaluated using the coefficient of determination of the calibration set $\left(R_{C}^{2}\right)$, mean squared error of calibration (MSEC), root mean square error of calibration (RMSEC), coefficient of determination of the prediction set $\left(R_{P}^{2}\right)$, mean squared error of prediction (MSEP), root mean square error of prediction (RMSEP), and residual predictive deviation (RPD). Models with higher $R_{C}^{2}$, $R_{P}^{2}$, and RPD values, together with lower MSEC, RMSEC, MSEP, and RMSEP values, were considered to have better predictive performance.

The main evaluation metrics were calculated as follows:

(3) $$R^{2}=1-\frac{\sum_{i=1}^{n}{\left(y_{i}-{\hat{y}}_{i}\right)}^{2}}{\sum_{i=1}^{n}(y_{i}-\overset{ˉ}{y}{)}^{2}}$$

(4) $$MSE=\frac{1}{n}\sum_{i=1}^{n}{\left(y_{i}-{\hat{y}}_{i}\right)}^{2}$$

(5) $$RMSE=\sqrt{\frac{1}{n}\sum_{i=1}^{n}{\left(y_{i}-{\hat{y}}_{i}\right)}^{2}}$$

(6) $$RPD=\frac{SD}{RMSEP}$$

where $y_{i}$ is the measured value of the ith sample; ${\hat{y}}_{i}$ is the predicted value; $\overset{ˉ}{y}$ is the mean measured value; n is the number of samples; and SD is the standard deviation of the measured values in the prediction set.

### 2.10. Statistical Analysis

Experimental data were expressed as the mean ± standard deviation. Differences among yam varieties were analyzed by one-way analysis of variance (ANOVA) using IBM SPSS Statistics 27. When significant differences were detected, post hoc multiple comparisons were performed at a significance level of *p* < 0.05. For each variety, Pearson correlation coefficients between the selected fresh-yam traits and dried-slice texture characteristics were calculated separately using MATLAB R2024a, based on 12 observations, and *p* < 0.05 was considered statistically significant. Because the adhesiveness values obtained from texture profile analysis were negative, their absolute values were used to represent dried-slice adhesion magnitude in the correlation analysis. Correlation heatmaps were generated using MATLAB. Spectral preprocessing, informative wavelength selection, and model development were performed using Python 3.8, while the other figures were prepared using Origin 2021.

## 3. Results and Discussion

### 3.1. Differences in Physicochemical Properties Among Yam Varieties

#### 3.1.1. Reducing Sugar Content

**Figure 2:**
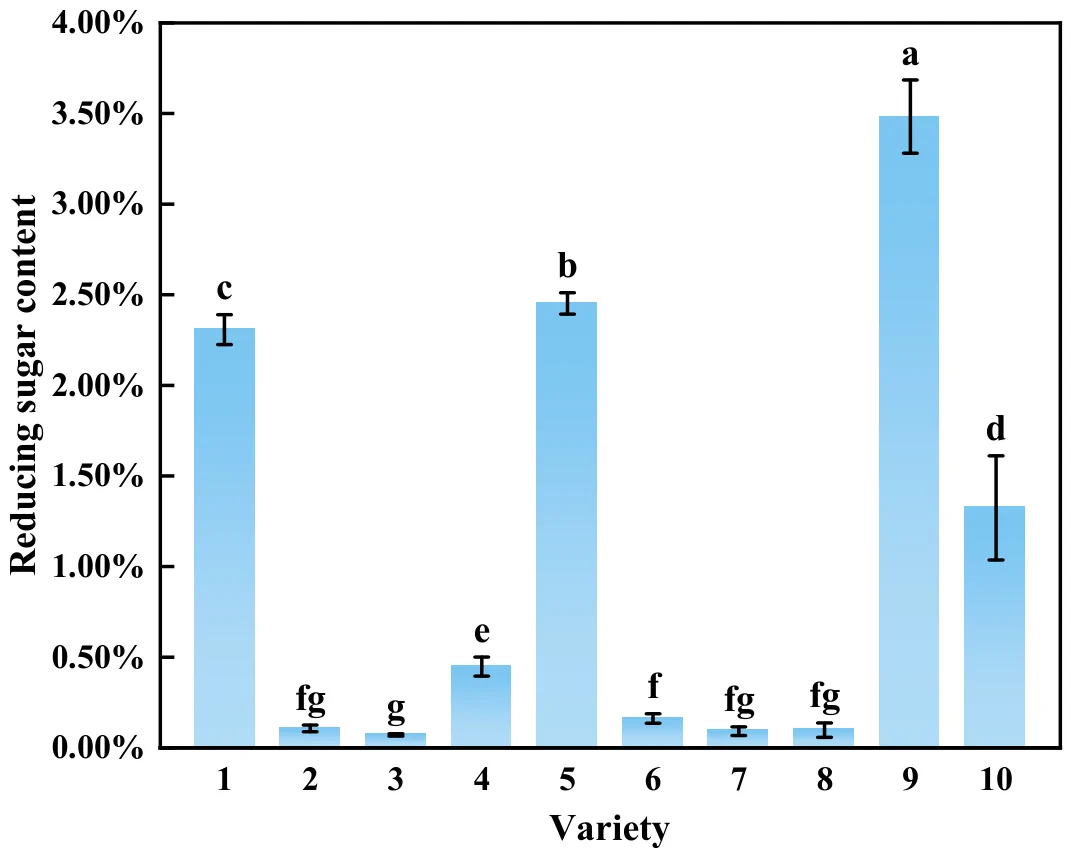
Reducing sugar contents of different yam varieties. Values are expressed as mean ± SD (*n* = 12). Different lowercase letters indicate significant differences among varieties (*p* < 0.05).

The reducing sugar contents of the ten yam varieties are shown in Figure 2. Significant differences were observed among varieties. Variety 9 had the highest reducing sugar content, reaching 3.48%, followed by variety 5 with 2.45%. In contrast, variety 3 showed the lowest value, at only 0.07%. The reducing sugar contents of varieties 2, 3, 7, and 8 were relatively low and did not differ significantly from each other.

The wide variation in reducing sugar content indicates clear differences in carbohydrate-related traits among the tested yam varieties. Since reducing sugar was one of the raw material indicators examined in this study, the observed variation indicates that yam variety should be considered when selecting raw materials for dried slice processing. Varieties with markedly different reducing sugar levels may not show the same processing suitability, even when prepared under the same drying conditions.

#### 3.1.2. Total Phenolic Content

**Figure 3:**
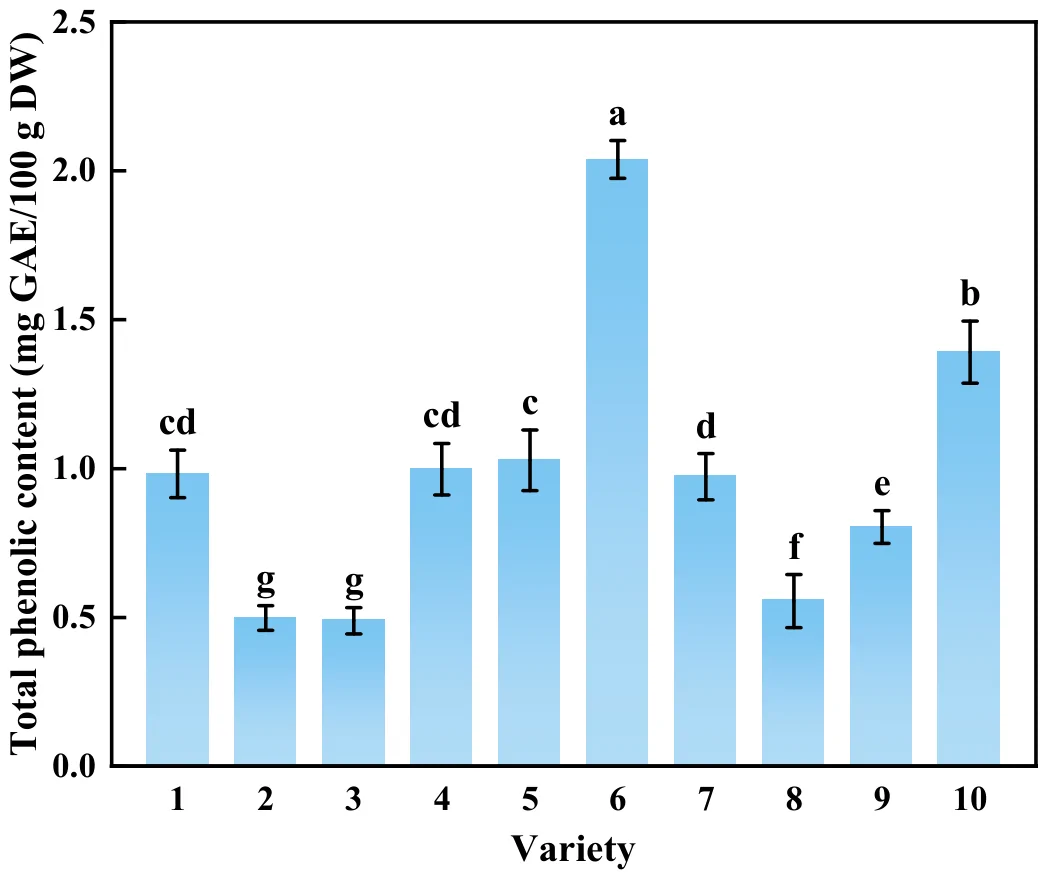
Total phenolic contents of different yam varieties. Values are expressed as mean ± SD (*n* = 12). Different lowercase letters indicate significant differences among varieties (*p* < 0.05).

The total phenolic contents of the ten yam varieties are shown in Figure 3. Significant differences were observed among varieties. Variety 6 showed the highest total phenolic content, reaching 2.04 mg GAE/100 g DW, followed by variety 10 with 1.39 mg GAE/100 g DW. Among the ten varieties, variety 3 had the lowest total phenolic content (0.49 mg GAE/100 g DW), and its value was close to that of variety 2.

Compared with reducing sugar content, the distribution of total phenolic content showed a different varietal pattern. Variety 6 had the highest total phenolic content but was not among the varieties with the highest reducing sugar content. This result suggests that different quality indicators should be considered together when evaluating yam raw materials. A single index is not sufficient to describe the overall suitability of a variety for dried slice processing.

### 3.2. Texture Properties of Fresh Yam Slices

**Figure 4:**
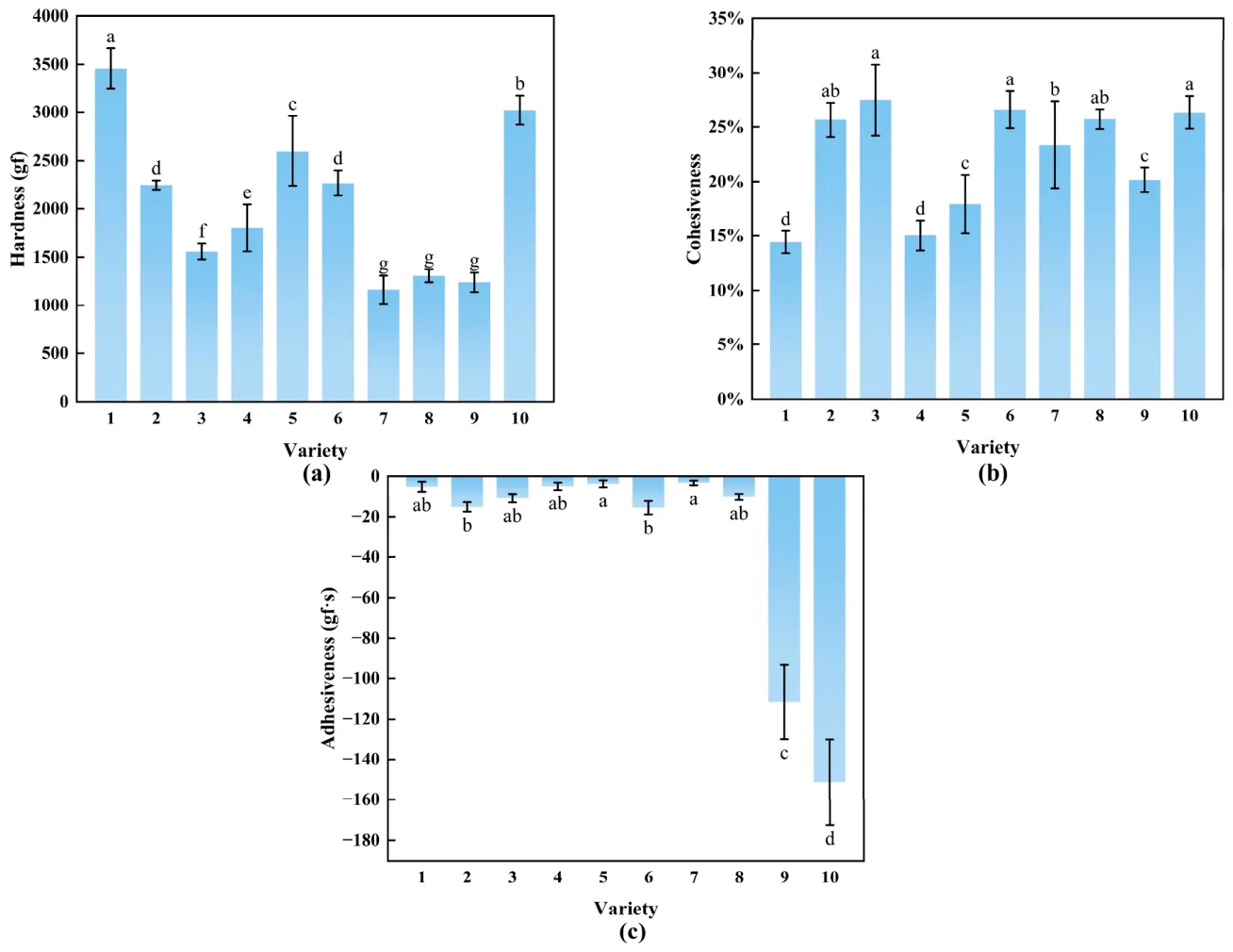
Texture properties of fresh yam slices from different varieties: (**a**) hardness; (**b**) cohesiveness; (**c**) adhesiveness. Cohesiveness is presented as a percentage. Values are expressed as mean ± SD (*n* = 12). Different lowercase letters indicate significant differences among varieties (*p* < 0.05).

The texture properties of fresh yam slices are shown in Figure 4. Hardness differed significantly among the ten yam varieties. Variety 1 had the highest hardness, with a mean value of 3455.42 gf, followed by variety 10 (3020.69 gf) and variety 5 (2600.20 gf). Variety 7 had the lowest hardness value (1163.23 gf), followed by varieties 9 and 8, and their hardness values were not significantly different.

Cohesiveness showed a different varietal distribution from hardness. Variety 3 had the highest mean cohesiveness (27.5%), followed by varieties 6 (26.6%) and 10 (26.3%). Varieties 8 and 2 also showed relatively high cohesiveness values (25.7% each). In contrast, variety 1 had the lowest cohesiveness (14.4%), followed closely by variety 4 (15.0%). Thus, higher hardness did not necessarily correspond to higher cohesiveness. This was particularly clear for variety 1, which had the highest hardness but relatively low cohesiveness.

Adhesiveness also differed significantly among varieties (*p* < 0.05) and showed a more uneven varietal pattern. In TPA, adhesiveness is expressed as a negative force–time area, with more negative values indicating stronger adhesion. Variety 10 showed the strongest adhesiveness, with a mean value of −151.31 gf·s, followed by variety 9 (−111.65 gf·s). In contrast, variety 7 showed the weakest adhesiveness, with a value closest to zero (−3.23 gf·s), followed by variety 5 (−3.70 gf·s).

Taken together with the differences in reducing sugar and total phenolic contents, the texture results showed clear varietal differences in the raw material characteristics of fresh yam before drying. The three texture parameters showed different varietal patterns. Hardness mainly represented tissue firmness, whereas cohesiveness and adhesiveness reflected different aspects of the sample response during TPA. Because hardness is a direct descriptor of tissue firmness, it was used as the texture variable in the subsequent hyperspectral prediction models. Taken together with the differences in reducing sugar and total phenolic contents, the texture results demonstrated clear varietal differences among the selected raw-material quality indicators.

### 3.3. Relationships Between Raw-Material Quality Indicators and Dried-Slice Texture Characteristics

**Figure 5:**
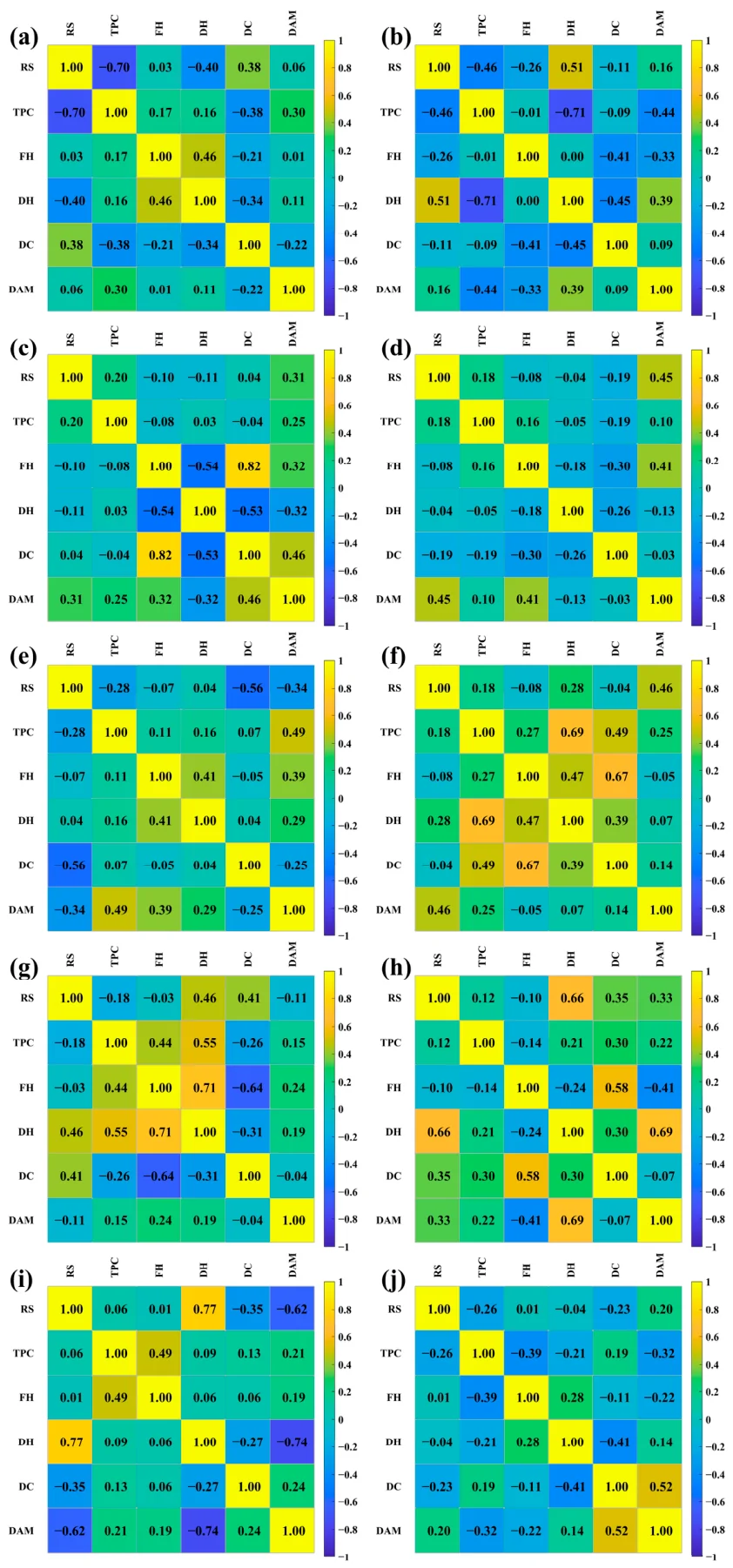
Pearson correlation matrices between selected raw-material quality indicators and dried-slice texture characteristics across ten Chinese yam varieties. (**a**–**j**) Varieties 1–10, respectively. RS, reducing sugar content; TPC, total phenolic content; FH, fresh-slice hardness; DH, dried-slice hardness; DC, dried-slice cohesiveness; DAM, dried-slice adhesion magnitude. Absolute adhesiveness values were used to represent adhesion magnitude in the correlation analysis. The variety numbers correspond to the yam varieties listed in Section 2.1.

The correlations between the selected raw-material quality indicators and dried-slice texture characteristics are presented in Figure 5. The correlation patterns differed among the ten varieties, and no single fresh-yam indicator showed a consistent relationship with dried-slice texture across all varieties.

Total phenolic content showed opposite relationships with dried-slice hardness in different varieties. In variety 2, total phenolic content was negatively correlated with dried-slice hardness (r = −0.71), whereas a positive correlation was observed in variety 6 (r = 0.69). These contrasting trends indicate that total phenolic content was not a consistent indicator of dried-slice hardness across varieties. Its relationship with the final texture may also be influenced by other compositional and structural characteristics of the raw material.

Fresh-slice hardness was more frequently associated with dried-slice cohesiveness than with dried-slice hardness. Positive correlations between fresh-slice hardness and dried-slice cohesiveness were observed in varieties 3, 6, and 8, with correlation coefficients of 0.82, 0.67, and 0.58, respectively. In contrast, a negative correlation was found in variety 7 (r = −0.64). Fresh-slice hardness was also positively correlated with dried-slice hardness in variety 7 (r = 0.71). These results suggest that greater initial firmness did not necessarily produce harder dried slices and that the relationship between fresh- and dried-slice texture varied among varieties.

Reducing sugar content was positively correlated with dried-slice hardness in varieties 8 and 9, with correlation coefficients of 0.66 and 0.77, respectively. In variety 9, reducing sugar content was also negatively correlated with dried-slice adhesion magnitude (r = −0.62). Similar relationships were not detected in the other varieties, indicating that the association between reducing sugar content and dried-slice texture was also variety dependent.

No significant correlations between the selected fresh-yam traits and dried-slice texture characteristics were detected in varieties 1, 4, 5, and 10. Taken together, the results show that dried-slice texture was associated with different raw material characteristics in different varieties. Therefore, the suitability of yam raw materials for dried-slice processing should be evaluated using multiple compositional and texture-related indicators rather than relying on a single parameter.

### 3.4. Spectral Characteristics and Preprocessing

**Figure 6:**
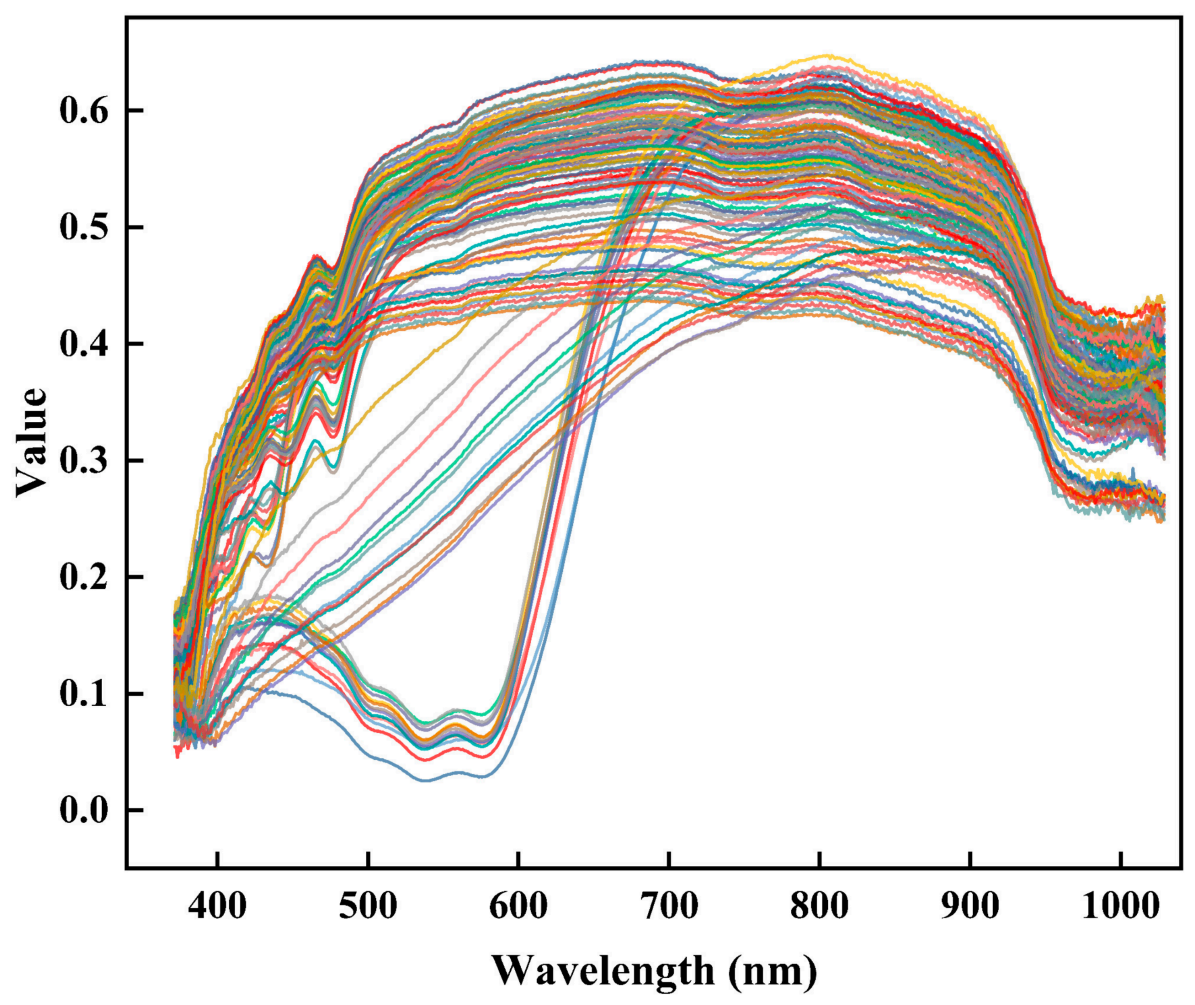
Original reflectance spectra of fresh yam slices in the wavelength range of approximately 371–1028 nm.

**Figure 7:**
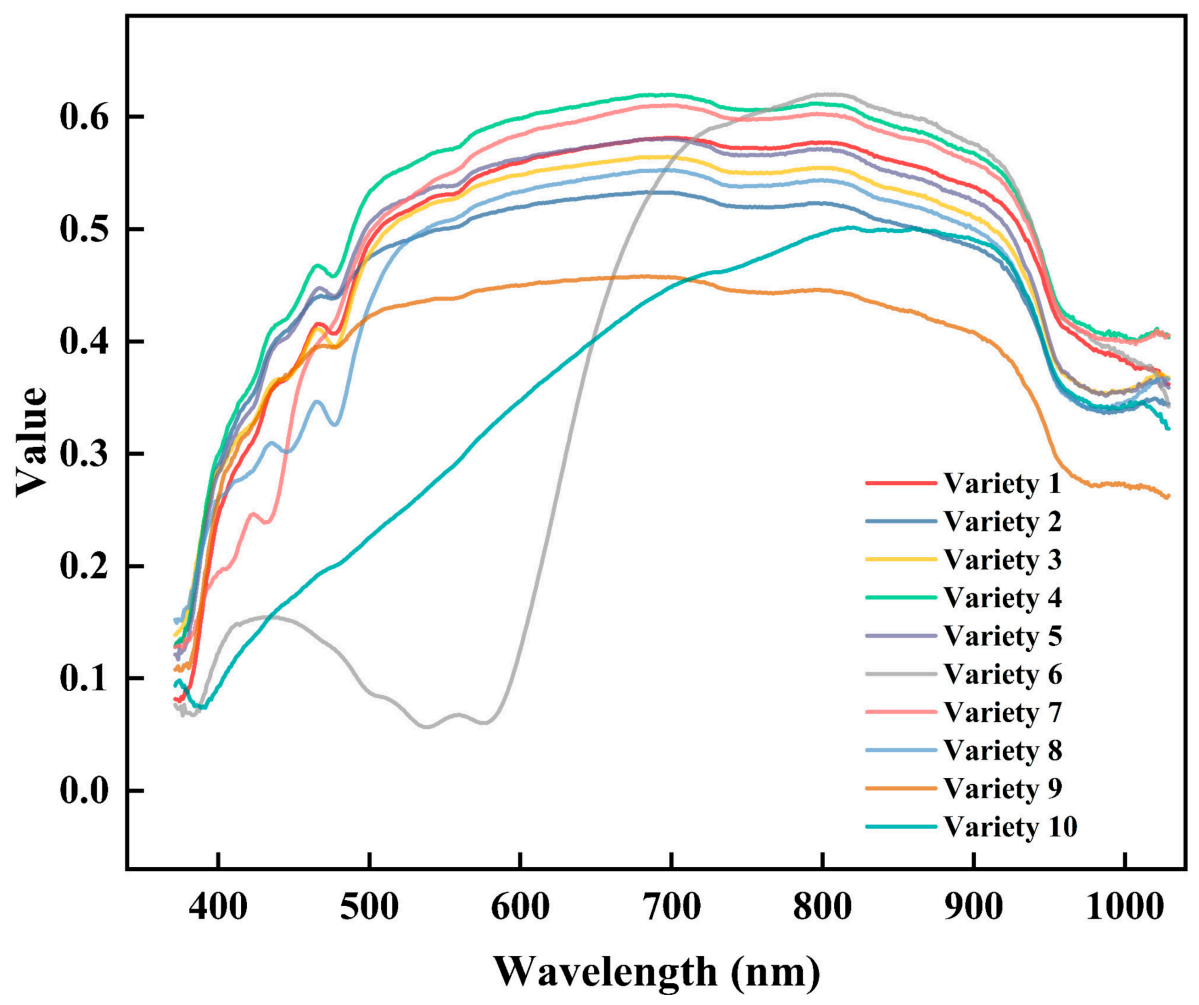
Mean reflectance spectra of the ten fresh yam varieties.

The original reflectance spectra of fresh yam slices were obtained in the wavelength range of approximately 371–1028 nm, with 616 spectral bands, as shown in Figure 6. The mean spectra of the ten yam varieties are shown in Figure 7. The spectra followed a broadly similar trend across the measured wavelength range, but the reflectance intensity varied among varieties. Varieties 1, 2, 3, 4, 5, 7, 8, and 9 showed relatively close spectral profiles, whereas varieties 6 and 10 differed more clearly from the others, particularly in the visible region. Since variety 6 was purple yam, its distinct response in this region was likely related to differences in surface colour and pigment absorption. The spectral difference observed in variety 10 may be associated with varietal differences in surface and tissue characteristics.

In general, the reflectance increased from the short-wavelength region toward the near-infrared region and then became relatively stable or slightly decreased at longer wavelengths. The lower reflectance in the visible region may be related to stronger absorption by colour-related components, while the near-infrared response is usually influenced by tissue structure, water status, and major chemical constituents. Therefore, the spectral differences among varieties may reflect the combined effects of surface appearance, internal structure, and composition. These differences were considered as spectral evidence of varietal variation, rather than direct proof of changes in any single component.

**Figure 8:**
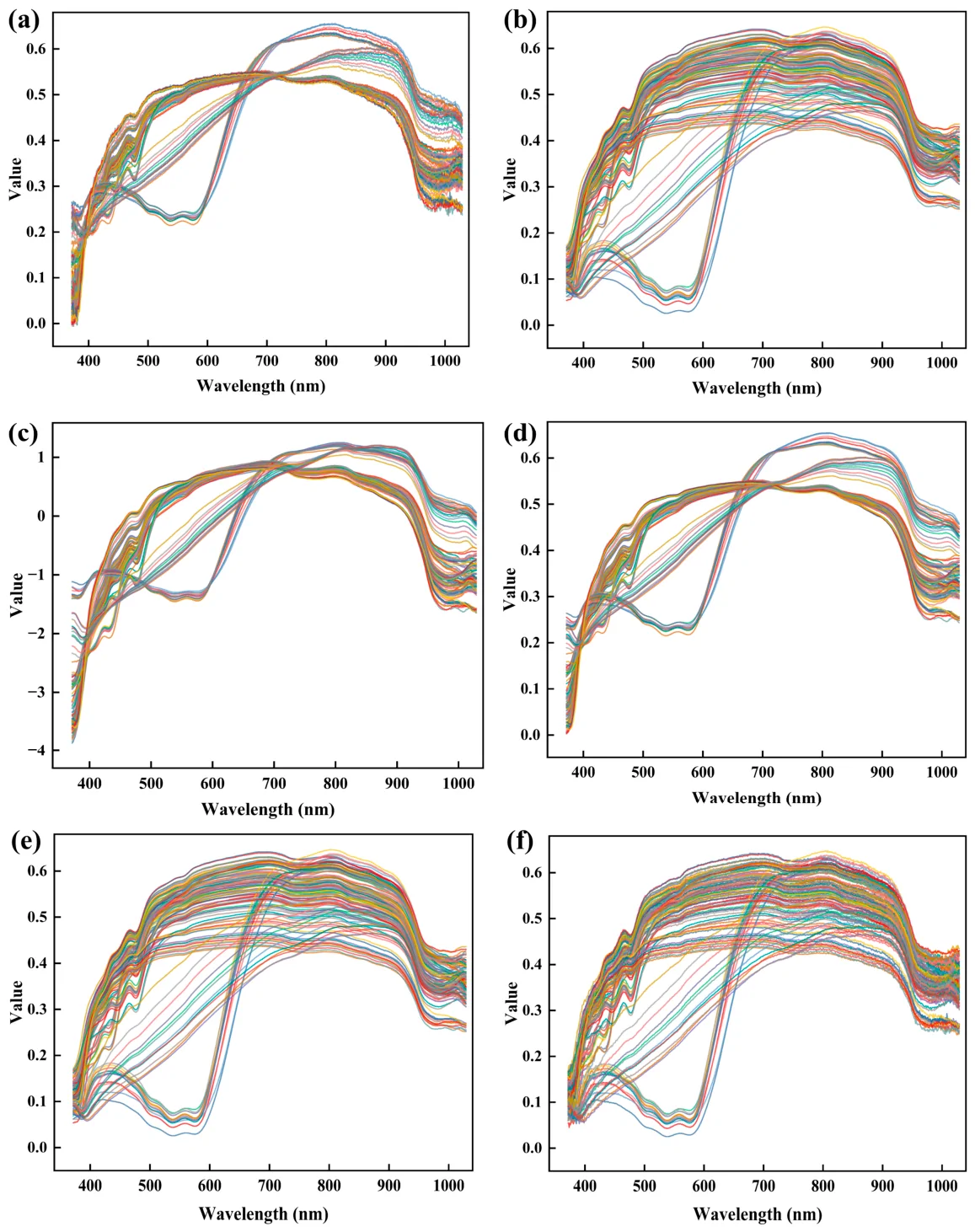
Representative spectra of fresh yam slices after different preprocessing methods: (**a**) MSC; (**b**) SG; (**c**) SG+SNV; (**d**) SG+MSC; (**e**) moving average smoothing; and (**f**) wavelet transformation.

The raw spectra showed baseline variation and local fluctuations, which may influence spectral consistency and quantitative modelling performance. To reduce these effects, different preprocessing methods were compared, including MSC, SNV, SG, SG + SNV, SG + MSC, moving average smoothing, first derivative, second derivative, wavelet transform, mean centralization, standardization, and min–max normalization. For clarity, only representative preprocessing results are displayed in Figure 8. SG smoothing reduced local fluctuations in the spectral curves, while MSC and SG+MSC reduced scattering-related baseline variation among samples. The combined SG+SNV treatment further improved spectral consistency while maintaining the main spectral profile. Moving average smoothing and wavelet transformation also reduced spectral fluctuations and retained the major spectral characteristics.

The effect of preprocessing was further evaluated using PLSR models for reducing sugar prediction, and the results are shown in Table 1. Among the tested preprocessing methods, SG+SNV gave the most suitable result for subsequent modelling, with $R_{C}^{2}$, $R_{P}^{2}$, RMSEP, and RPD values of 0.9507, 0.9076, 0.4425, and 2.1625, respectively. Although the first- and second-derivative spectra produced high fitting results in the calibration set, their prediction-set performance was less stable. The second derivative, for instance, resulted in a high $R_{C}^{2}$ but a much lower $R_{P}^{2}$, higher RMSEP, and lower RPD, indicating poor generalization. Based on these results, SG+SNV was selected as the preprocessing method for subsequent feature selection and model development.

### 3.5. Dimensionality Reduction and Feature Selection

Dimensionality reduction and feature selection were performed before regression modelling to reduce the number of spectral variables. The preprocessed spectra contained 616 bands. In this study, PCA, CARS, and SPA were used for spectral variable processing. PCA extracted principal components from the preprocessed full spectra, whereas CARS and SPA selected characteristic wavelengths from the preprocessed spectral variables.

The retained variables differed among the three methods and among the predicted quality indicators. For reducing sugar content, PCA retained 29 principal components, while CARS and SPA selected 215 and 27 wavelengths, respectively. For total phenolic content, PCA retained 24 principal components, and CARS and SPA selected 272 and 29 wavelengths, respectively. For hardness, PCA retained 19 principal components, whereas CARS and SPA selected 343 and 11 wavelengths, respectively. These differences indicate that the spectral variables used for model construction were dependent on both the target trait and the variable selection method.

PCA reduced the dimensionality of the preprocessed full spectra through principal component extraction, but the extracted variables did not correspond to individual wavelength positions. In contrast, CARS and SPA retained wavelength variables directly from the preprocessed spectra. CARS retained more wavelengths than SPA for all three indicators, especially for total phenolic content and hardness. SPA produced more compact wavelength subsets. The retained principal components or selected wavelengths were then used as input variables for the regression models.

### 3.6. Prediction of Reducing Sugar Content

**Figure 9:**
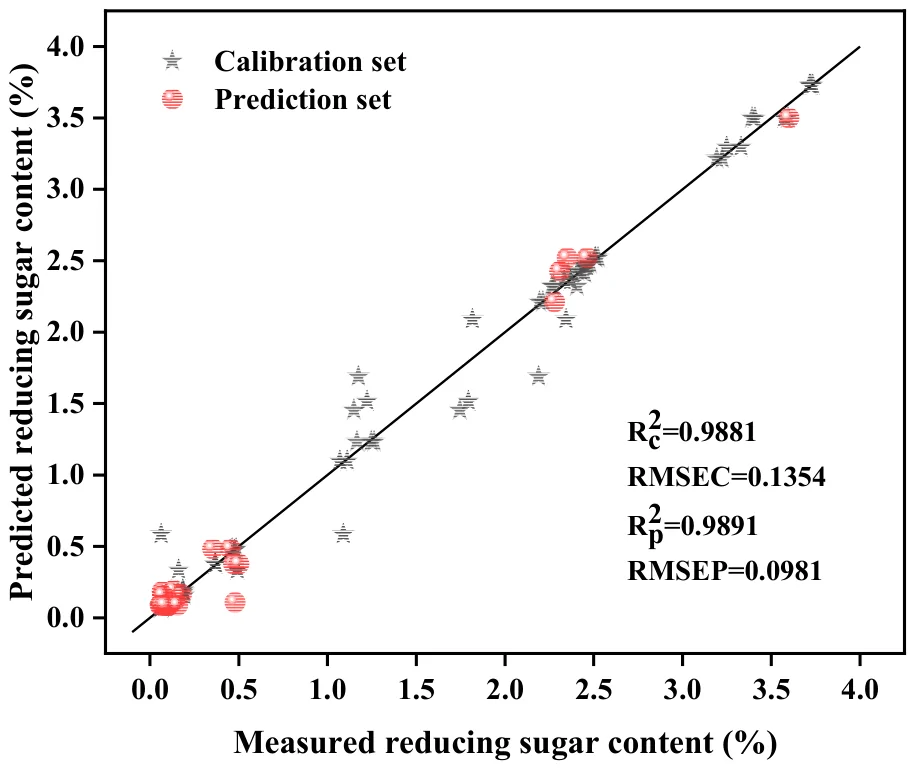
Measured and predicted reducing sugar contents using the PCA-DTR model.

The prediction results for reducing sugar content are shown in Table 2, and the scatter plot of the best-performing model is shown in Figure 9. The prediction performance differed among the combinations of variable processing methods and regression models. Among all tested combinations, the PCA-DTR model showed the best performance, with $R_{C}^{2}$, RMSEC, $R_{P}^{2}$, RMSEP, and RPD values of 0.9881, 0.1354, 0.9891, 0.0981, and 9.7535, respectively.

Compared with the models based on CARS- and SPA-selected variables, the PCA-based models generally gave better prediction results for reducing sugar content. This indicates that the spectral information related to reducing sugar was well represented by the principal components extracted from the preprocessed full spectra. The high $R_{P}^{2}$ and low RMSEP of the PCA-DTR model showed good agreement between measured and predicted values in the prediction set.

These results suggest that PCA combined with DTR was suitable for predicting reducing sugar content in fresh yam slices. Therefore, PCA-DTR was selected as the optimal model for reducing sugar content prediction in this study.

### 3.7. Prediction of Total Phenolic Content

**Figure 10:**
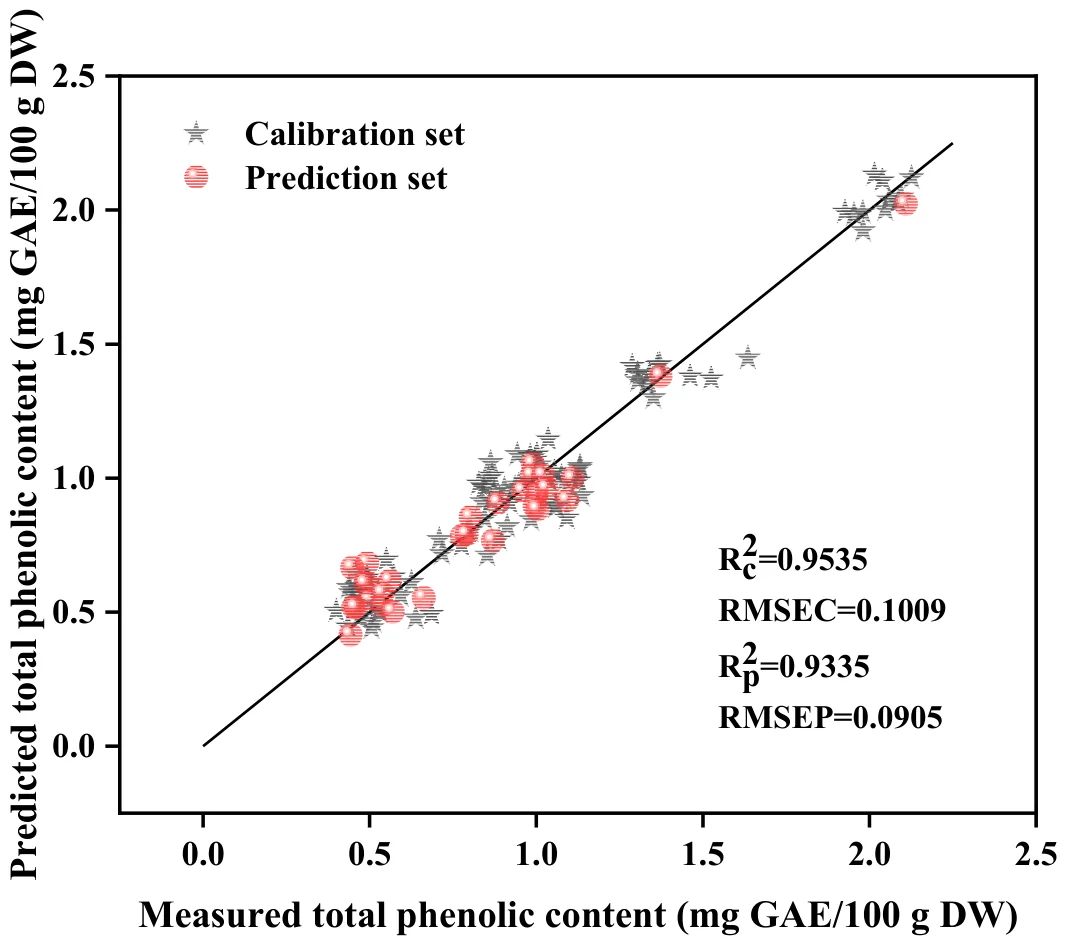
Measured and predicted total phenolic contents using the CARS-PLSR model.

The prediction results for total phenolic content are shown in Table 3, and the scatter plot of the best-performing model is shown in Figure 10. The performance of the models differed among the variable processing methods and regression algorithms. Among all tested combinations, the CARS-PLSR model gave the best prediction result, with $R_{C}^{2}$, RMSEC, $R_{P}^{2}$, RMSEP, and RPD values of 0.9535, 0.1009, 0.9335, 0.0905, and 3.9438, respectively.

Compared with PCA and SPA, CARS provided better variable inputs for total phenolic content prediction. The CARS-PLSR model showed good agreement between measured and predicted values in the prediction set, as reflected by its relatively high $R_{P}^{2}$ and low RMSEP. This result indicates that the wavelengths retained by CARS contained useful information related to total phenolic content.

Among the regression models, PLSR performed well when combined with CARS-selected wavelengths. This suggests that the relationship between the selected spectral variables and total phenolic content could be effectively described by a latent-variable regression model. Therefore, CARS-PLSR was selected as the optimal model for total phenolic content prediction in this study.

It should be clarified that the hyperspectral data were acquired from fresh yam slices before drying, whereas the reducing sugar and TPC reference values were determined from the corresponding samples after drying and grinding. In this study, drying and grinding were used as analytical sample-preparation procedures for chemical determination rather than to define the prediction targets as the composition of the final dried product. The use of dried or freeze-dried ground yam material for chemical analysis has also been reported in previous studies. For example, Guo et al. [16] used freeze-dried and ground Chinese purple yam material for phenolic analysis, while Zhang et al. [33] dried raw and processed Chinese yam samples at 60 °C before grinding and determining reducing sugar content according to the Chinese national standard with results expressed on a dry-weight basis.

Nevertheless, thermal treatment may alter the absolute contents of reducing sugars and phenolic compounds, and drying conditions have been reported to affect bioactive components in yam [9]. Therefore, the RS and TPC values used in this study should not be interpreted as the native concentrations in untreated fresh tissue. Rather, they represent chemical reference values obtained using the specified analytical procedures. Because each hyperspectral observation was paired with a reference measurement from the corresponding tuber and all samples were subjected to the same sample-preparation and analytical procedures, these values provided consistent targets for model development.

The prediction-set results indicate that the spectral information acquired before drying was associated with these reference measurements under the conditions examined in this study. Similar calibration strategies have been applied in food quality assessment, where hyperspectral measurements acquired from raw materials were correlated with reference values obtained using conventional destructive laboratory procedures [21]. These studies demonstrate the feasibility of establishing quantitative relationships between hyperspectral information and chemically determined reference values through multivariate modelling [34]. Therefore, fresh-slice HSI shows potential for rapid screening of yam raw materials before processing.

### 3.8. Prediction of Hardness

**Figure 11:**
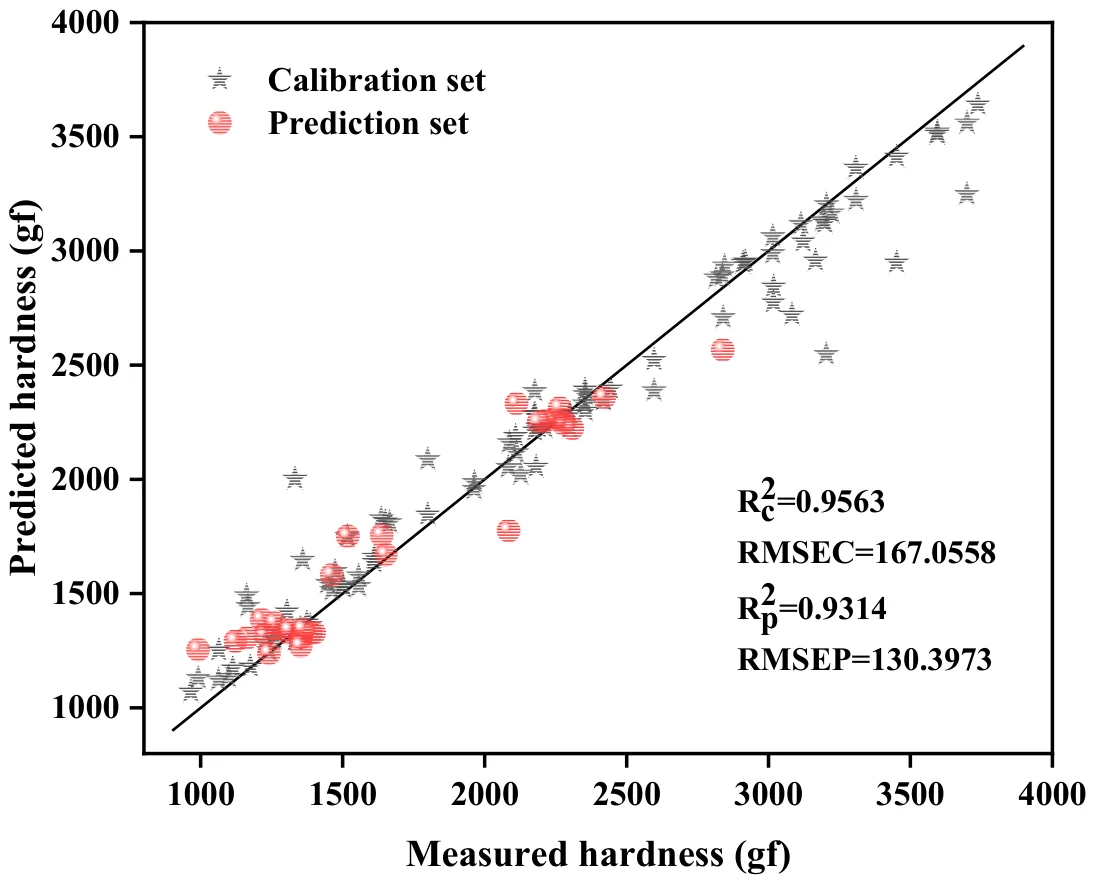
Measured and predicted hardness values using the CARS-RFR model.

The prediction results for hardness are summarized in Table 4, and the scatter plot of the best-performing model is shown in Figure 11. The prediction performance varied among different combinations of variable processing methods and regression models. Among all tested combinations, the CARS-RFR model achieved the best result, with $R_{C}^{2}$, RMSEC, $R_{P}^{2}$, RMSEP, and RPD values of 0.9563, 167.0558, 0.9314, 130.3973, and 3.8820, respectively.

Compared with PCA and SPA, CARS provided more suitable wavelength variables for hardness prediction. The CARS-RFR model showed a relatively high $R_{P}^{2}$ and low RMSEP in the prediction set, indicating good agreement between measured and predicted hardness values. This result suggests that the wavelengths retained by CARS contained useful information related to the texture differences among fresh yam slices.

Among the regression models, RFR performed best when combined with CARS-selected wavelengths. Since hardness is closely related to tissue structure and mechanical resistance, its relationship with spectral information may not be strictly linear. The better performance of RFR indicates that a nonlinear ensemble model was suitable for describing the relationship between spectral variables and hardness. Therefore, CARS-RFR was selected as the optimal model for hardness prediction in this study.

Overall, the best-performing model differed among the three quality indicators. PCA-DTR gave the best result for reducing sugar content, CARS-PLSR for total phenolic content, and CARS-RFR for hardness. This difference suggests that the spectral variables and regression methods suitable for one quality trait were not necessarily optimal for another. Therefore, the prediction model should be selected according to the target indicator rather than using the same model structure for all traits.

## 4. Conclusions

This study compared the major raw material quality traits of ten Chinese yam varieties, examined their relationships with dried-slice texture characteristics, and evaluated HSI-based models for the rapid assessment of key quality indicators. Clear varietal differences were observed in reducing sugar content, total phenolic content, and texture-related properties. Variety 9 had the highest reducing sugar content, variety 6 had the highest total phenolic content, and variety 1 showed the highest fresh-slice hardness, confirming substantial variation in raw material quality among the tested varieties.

The relationships between raw-material traits and dried-slice texture characteristics also differed among varieties. Total phenolic content was correlated with dried-slice hardness in varieties 2 and 6, although the correlation directions were opposite. Fresh-slice hardness was more frequently associated with dried-slice cohesiveness, while reducing sugar content was positively correlated with dried-slice hardness in varieties 8 and 9. Therefore, the suitability of yam raw materials for dried-slice processing cannot be adequately assessed using a single compositional or texture-related parameter.

HSI acquired from fresh yam slices was used to predict the reference values of reducing sugar content and total phenolic content obtained using the specified analytical procedures, together with directly measured fresh-slice hardness. These results demonstrate the potential of fresh-slice HSI for rapid screening and assessment of yam raw materials before processing. However, the predicted values of reducing sugar content and total phenolic content should be interpreted as chemical reference values obtained using the specified analytical procedures rather than the native concentrations in untreated fresh tissues. SG+SNV was selected for spectral preprocessing. Following dimensionality reduction and informative wavelength selection using PCA, CARS, and SPA, the best-performing models were PCA-DTR for reducing sugar content, CARS-PLSR for total phenolic content, and CARS-RFR for hardness. The corresponding prediction-set coefficients of determination ($R_{P}^{2}$), RMSEP, and RPD values were 0.9891, 0.0981%, and 9.7535 for reducing sugar content; 0.9335, 0.0905 mg GAE/100 g DW, and 3.9438 for total phenolic content; and 0.9314, 130.3973 gf, and 3.8820 for hardness, respectively. The variation in the best-performing model combinations shows that spectral variable processing and regression methods need to be selected according to the quality indicator being predicted.

In summary, HSI combined with appropriate spectral preprocessing, variable selection, and regression modelling showed good potential for the rapid assessment of quality traits relevant to dried yam slice processing. The results provide a basis for screening and selecting Chinese yam raw materials before drying.

## Figures and Tables

**Table 1:** Comparison of models using different spectral preprocessing methods.

Preprocessing Method	Calibration Set			Prediction Set			RPD
	$R_{C}^{2}$	MSEC	RMSEC (%)	$R_{P}^{2}$	MSEP	RMSEP (%)	
MSC	0.9070	0.1202	0.3467	0.8610	0.2945	0.5427	1.7632
SNV	0.9205	0.1027	0.3205	0.8780	0.2585	0.5084	1.8822
SG	0.8050	0.2519	0.5019	0.8134	0.3952	0.6287	1.5220
SG+SNV	0.9507	0.0636	0.2523	0.9076	0.1958	0.4425	2.1625
SG+MSC	0.8972	0.1224	0.3490	0.8821	0.2395	0.4893	1.9557
moving average	0.8619	0.1780	0.4223	0.8334	0.3530	0.5942	1.6104
First derivative	0.9620	0.0491	0.2215	0.8582	0.3005	0.5481	1.7458
Second derivative	0.9974	0.0034	0.0583	0.6299	0.7842	0.8855	1.0806
Wavelet transform	0.8018	0.2560	0.5059	0.8074	0.4081	0.6388	1.4980
mean centralization	0.8070	0.2493	0.4993	0.8138	0.3944	0.6280	1.5237
standardization	0.8025	0.3482	0.5901	0.8324	0.3453	0.5876	1.6285
min–max normalization	0.9232	0.0915	0.3025	0.7294	0.5496	0.7414	1.2907

**Table 2:** Prediction performance of models based on different spectral variable processing methods for reducing sugar content.

Variable Processing Method	Number of Variables	Model	Calibration Set			Prediction Set			RPD
			$R_{C}^{2}$	MSEC	RMSEC (%)	$R_{P}^{2}$	MSEP	RMSEP (%)	
PCA	29	PLSR	0.9346	0.1004	0.3169	0.8738	0.1117	0.3343	2.8626
		SVR	0.9999	0.0001	0.0100	0.7533	0.2183	0.4672	2.0479
		RFR	0.9545	0.0699	0.2644	0.6828	0.2807	0.5298	1.8059
		DTR	0.9881	0.0183	0.1354	0.9891	0.0096	0.0981	9.7535
CARS	215	PLSR	0.9694	0.0470	0.2168	0.8999	0.0886	0.2977	3.2143
		SVR	0.7015	0.4583	0.6770	0.7020	0.2637	0.5136	1.8632
		RFR	0.9653	0.0533	0.2308	0.8458	0.1365	0.3695	2.5899
		DTR	0.9851	0.0229	0.1512	0.9607	0.0347	0.1864	5.1337
SPA	27	PLSR	0.8411	0.2440	0.4940	0.5447	0.4030	0.6348	1.5073
		SVR	0.7768	0.3427	0.5854	0.7252	0.2432	0.4931	1.9404
		RFR	0.9637	0.0557	0.2360	0.7929	0.1833	0.4281	2.2350
		DTR	0.9943	0.0088	0.0937	0.7778	0.1967	0.4435	2.1576

**Table 3:** Prediction performance of models based on different spectral variable processing methods for total phenolic content.

Variable Processing Method	Number of Variables	Model	Calibration Set			Prediction Set			RPD
			$R_{C}^{2}$	MSEC	RMSEC (mg GAE/100 g DW)	$R_{P}^{2}$	MSEP	RMSEP (mg GAE/100 g DW)	
PCA	24	PLSR	0.9447	0.0121	0.1099	0.9066	0.0115	0.1072	3.3278
		SVR	0.9995	0.0001	0.0100	0.6888	0.0383	0.1957	1.8233
		RFR	0.9688	0.0068	0.0826	0.7598	0.0296	0.1719	2.0752
		DTR	0.9934	0.0014	0.0380	0.8158	0.0227	0.1505	2.3701
CARS	272	PLSR	0.9535	0.0102	0.1009	0.9335	0.0082	0.0905	3.9438
		SVR	0.9596	0.0088	0.0940	0.9303	0.0086	0.0926	3.8528
		RFR	0.9898	0.0022	0.0473	0.9242	0.0093	0.0966	3.6941
		DTR	0.9965	0.0008	0.0275	0.9180	0.0101	0.1004	3.5521
SPA	29	PLSR	0.9319	0.0149	0.1220	0.8880	0.0138	0.1174	3.0396
		SVR	0.9706	0.0064	0.0801	0.9327	0.0083	0.0910	3.9203
		RFR	0.9895	0.0023	0.0479	0.9207	0.0098	0.0988	3.6117
		DTR	0.9972	0.0006	0.0248	0.8949	0.0129	0.1137	3.1374

**Table 4:** Prediction performance of models based on different spectral variable processing methods for hardness.

Variable Processing Method	Number of Variables	Model	Calibration Set			Prediction Set			RPD
			$R_{C}^{2}$	MSEC	RMSEC (gf)	$R_{P}^{2}$	MSEP	RMSEP (gf)	
PCA	19	PLSR	0.8560	91,875.4454	303.1096	0.6417	88,760.4487	297.9269	1.6991
		SVR	0.0511	605,380.7504	778.0622	−0.8881	467,683.5018	683.8739	0.7402
		RFR	0.9579	26,866.8005	163.9110	0.7613	59,112.9740	243.1316	2.0820
		DTR	0.9903	6187.4802	78.6605	0.6591	84,429.9586	290.5683	1.7421
CARS	343	PLSR	0.8691	83,506.2602	288.9745	0.6778	79,809.7796	282.5062	1.7918
		SVR	0.0617	598,634.3345	773.7146	−0.7562	435,018.0080	659.5589	0.7675
		RFR	0.9563	27,907.6319	167.0558	0.9314	17,003.4641	130.3973	3.8820
		DTR	0.9939	3912.2291	62.5478	0.7330	66,137.3599	257.1718	1.9683
SPA	11	PLSR	0.7601	153,073.5263	391.2461	0.6423	88,599.3175	297.6564	1.7006
		SVR	0.0682	594,475.0489	771.0221	−0.7372	430,306.6532	655.9776	0.7717
		RFR	0.9822	11,333.7645	106.4602	0.9233	18,996.6480	137.8283	3.6727
		DTR	0.9944	3550.0537	59.5823	0.4417	138,282.8831	371.8641	1.3613

## Data Availability

The data presented in this study are available on request from the corresponding author.

## Abbreviations

The following abbreviations are used in this manuscript:

PCA	Principal component analysis
DTR	Decision tree regression
CARS	Competitive adaptive reweighted sampling
PLSR	Partial least squares regression
RFR	Random forest regression
R_p_^2^	Coefficient of determination of the prediction set
RMSEP	Root mean square error of prediction
RPD	Residual predictive deviation
HSI	Hyperspectral imaging
SPA	Successive projections algorithm
SVR	Support vector regression
DNS	3,5-Dinitrosalicylic acid
TPC	Total phenolic content
GAE	Gallic acid equivalent
DW	Dry weight
TPA	Texture profile analysis
EMCCD	Electron-multiplying charge-coupled device
ROI	Region of interest
MSC	Multiplicative scatter correction
SNV	Standard normal variate
SG	Savitzky–Golay
SPXY	Sample set partitioning based on joint X–Y distances
$R_{C}^{2}$	Coefficient of determination of the calibration set
MSEC	Mean squared error of calibration
RMSEC	Root mean square error of calibration
MSEP	Mean squared error of prediction
$R_{P}^{2}$	Coefficient of determination of the prediction set
RMSEP	Root mean square error of prediction
RPD	Residual predictive deviation
SD	Standard deviation
ANOVA	Analysis of variance
